# A Metagenome-Wide Association Study of Gut Microbiome in Patients With Multiple Sclerosis Revealed Novel Disease Pathology

**DOI:** 10.3389/fcimb.2020.585973

**Published:** 2020-12-11

**Authors:** Toshihiro Kishikawa, Kotaro Ogawa, Daisuke Motooka, Akiko Hosokawa, Makoto Kinoshita, Ken Suzuki, Kenichi Yamamoto, Tatsuo Masuda, Yuki Matsumoto, Takuro Nii, Yuichi Maeda, Shota Nakamura, Hidenori Inohara, Hideki Mochizuki, Tatsusada Okuno, Yukinori Okada

**Affiliations:** ^1^Department of Statistical Genetics, Osaka University Graduate School of Medicine, Suita, Japan; ^2^Department of Otorhinolaryngology–Head and Neck Surgery, Osaka University Graduate School of Medicine, Suita, Japan; ^3^Department of Neurology, Osaka University Graduate School of Medicine, Suita, Japan; ^4^Department of Neurology, Japan Community Health care Organization (JCHO) Hoshigaoka Medical Center, Hirakata, Japan; ^5^Department of Infection Metagenomics, Research Institute for Microbial Diseases, Osaka University, Suita, Japan; ^6^Department of Neurology, Suita Municipal Hospital, Suita, Japan; ^7^Department of Pediatrics, Osaka University Graduate School of Medicine, Suita, Japan; ^8^Department of Obstetrics and Gynecology, Osaka University Graduate School of Medicine, Suita, Japan; ^9^Department of Respiratory Medicine and Clinical Immunology, Osaka University Graduate School of Medicine, Suita, Japan; ^10^Laboratory of Immune Regulation, Department of Microbiology and Immunology, Osaka University Graduate School of Medicine, Suita, Japan; ^11^Integrated Frontier Research for Medical Science Division, Institute for Open and Transdisciplinary Research Initiatives, Osaka University, Suita, Japan; ^12^Laboratory of Statistical Immunology, Immunology Frontier Research Center (WPI-IFReC), Osaka University, Suita, Japan

**Keywords:** metagenome shotgun sequencing, genome-wide association study, dysbiosis, gut microbiome, multiple sclerosis

## Abstract

While microbiome plays key roles in the etiology of multiple sclerosis (MS), its mechanism remains elusive. Here, we conducted a comprehensive metagenome-wide association study (MWAS) of the relapsing-remitting MS gut microbiome (*n*_case_ = 26, *n*_control_ = 77) in the Japanese population, by using whole-genome shotgun sequencing. Our MWAS consisted of three major bioinformatic analytic pipelines (phylogenetic analysis, functional gene analysis, and pathway analysis). Phylogenetic case-control association tests showed discrepancies of eight clades, most of which were related to the immune system (false discovery rate [FDR] < 0.10; e.g., *Erysipelatoclostridium_sp*. and *Gemella morbillorum*). Gene association tests found an increased abundance of one putative dehydrogenase gene (Clo1100_2356) and one ABC transporter related gene (Mahau_1952) in the MS metagenome compared with controls (FDR < 0.1). Molecular pathway analysis of the microbiome gene case-control comparisons identified enrichment of multiple Gene Ontology terms, with the most significant enrichment on cell outer membrane (*P* = 1.5 × 10^−7^). Interaction between the metagenome and host genome was identified by comparing biological pathway enrichment between the MS MWAS and the MS genome-wide association study (GWAS) results (i.e., MWAS-GWAS interaction). No apparent discrepancies in alpha or beta diversities of metagenome were found between MS cases and controls. Our shotgun sequencing-based MWAS highlights novel characteristics of the MS gut microbiome and its interaction with host genome, which contributes to our understanding of the microbiome’s role in MS pathophysiology.

## Introduction

The human microbiome is fingerprint of the pathogenesis and therapeutic effect of human complex diseases such as metabolic diseases, immune diseases, and cancer, as well as inflammatory bowel diseases (Forslund et al., 2015; Franzosa et al., 2019; Yachida et al., 2019; Kishikawa et al., 2020a). As illustrated in the word “brain-gut axis,” central nervous diseases are closely related to gut microbiome, and the disease mechanism in this context has been elucidated (Cryan et al., 2019). For example, Benakis *et al*. reported that effector T cells transferred from the gut to the brain after stroke enhanced ischemic neuroinflammation by secreting IL-17 (Benakis et al., 2016).

Multiple sclerosis (MS) is the most prevalent chronic immune-related inflammatory disease of the central nervous system, which devastates global health with socioeconomic burdens (Okuno et al., 2015; Reich et al., 2018). The prevalence rate is 50–300 per 100,000 people, with an estimated 2–3 million people globally affected with multiple sclerosis (Thompson et al., 2018). Genome-wide association studies (GWAS) and further fine mapping have discovered many MS-associated genomic regions so far and a part of its pathogenesis has been elucidated (International Multiple Sclerosis Genetics Consortium, 2019; Ogawa et al., 2019). Of note, the number of patients affected with MS in Japan has increased approximately four times in the last 30 years (Osoegawa et al., 2009), which indicates that there exists an emerging risk of developing MS other than the genetic factor. Gut microbiome is considered as one of the candidate risk factors of MS. So far, multiple MS-associated bacteria have been reported, and the biological effects of those bacteria on MS etiology have been demonstrated by using disease models such as experimental autoimmune encephalomyelitis (EAE) (Jangi et al., 2016; Berer et al., 2017; Cekanaviciute et al., 2017).

There exist two major analytical approaches utilizing next generation sequencing technologies in the field of microbiome studies; the classical one is 16S ribosomal RNA (rRNA) sequencing dealing with a part of the microbiome, and the advanced one is whole-genome shotgun sequencing (Ranjan et al., 2016). 16S rRNA sequencing can detect only the relative abundance of each taxon, not the biological functions. The taxonomic assessment is limited at the genus level, and less precise at the species level. On the other hand, whole-genome shotgun sequencing can detect the genomic composition of the microbiome with high resolution at the species level (not only bacteria but also archaea, fungi, and viruses) without bias induced from PCR and individual differences in the number of 16S rRNA. In addition, this novel method has another benefit of analyzing microbiome’s biological functional features, by conducting metagenome-wide association study (MWAS; (Kishikawa et al., 2020a; Kishikawa et al., 2020b; Zhu et al., 2020). While MWAS is a powerful to disentangle disease-related pathophysiology of microbiome, requirements of relatively high costs, computational resources to analyze large datasets of next generation sequencing, and complicated data analysis protocols have hampered its application. There exist few MWAS of MS based on whole-genome shotgun sequencing yet (Ventura et al., 2019).

Here, we report a comprehensive MWAS of the gut microbiome in a relapsing-remitting MS (RRMS) case-control cohort of the Japanese population. We carried out whole-genome shotgun sequencing of 103 fecal samples (26 individuals with MS and 77 healthy controls). Our MWAS consisted of three major bioinformatic analytic techniques (phylogenetic analysis, functional gene analysis, and pathway analysis), which allowed us to comprehensively grasp case-control disparity in the gut metagenome. We also compared the pathway enrichment of the gut microbiome MWAS and that of the host GWAS in MS to evaluate the link between the gut metagenome and the human germline genome (i.e., MWAS-GWAS interaction).

## Methods

### Patient Participation

We enrolled 28 RRMS patients at Osaka University Hospital. MS patients were diagnosed according to the McDonald 2010 criteria (Polman et al., 2011). The 77 healthy controls were enrolled at Osaka University Graduate School of Medicine. Healthy controls had no personal history of immune-related diseases and treatment with antibiotics for at least one month prior to sampling. Exclusion criteria for both groups were as follows: (i) under 20 years old, (ii) extreme diets (e.g., strict vegetarians), or (iii) treatment with antibiotics for at least 1 month prior to sampling. All the subjects provided written informed consent before participation. The study protocol was approved by the ethical committees of Osaka University and related medical institutions.

### Sample Collection and DNA Extraction

For patients, fecal samples had been immediately frozen after production in an insulated container for storage at −20°C and subsequently stored at −80°C within 24 h after production. For healthy controls, samples were stored at −80°C within 6 h after production. Bacterial DNA was extracted according to a previously described method (Maeda et al., 2016; Okumura et al., 2016). Briefly, RNAlater (Ambion) was added to make 10-fold dilutions of homogenates. Three hundred µl of sodium dodecyl sulfate–Tris solution, 0.3 g glass beads (diameter 0.1 mm) (BioSpec), and 500 µl EDTA-Tris-saturated phenol were added to the suspension, and the mixture was vortexed vigorously using a FastPrep-24 (MP Biomedicals) at 5.0 power level for 30 s. After centrifugation at 20,000 g for 5 min at 4°C, 400 µl of supernatant was collected. Subsequently, phenol-chloroform extraction was performed, and 250 µl of supernatant was subjected to isopropanol precipitation. Finally, DNAs were suspended in 200 µl EDTA-Tris buffer and stored at −80°C.

### Whole-Genome Shotgun Sequencing

A shotgun sequencing library was constructed using the KAPA Hyper Prep Kit (KAPA Biosystems) and 150 bp paired-end reads were generated on a HiSeq 3000 (average 7.3 Gb per sample). The sequence reads were converted to FASTQ format using bcl2fastq (version 2.19).

#### Quality Control (QC) of Sequencing Reads and Samples

We applied a series of QC steps to maximize the quality of the datasets. The main QC steps were: (i) trimming of low-quality bases, (ii) identification and masking of human reads, and (iii) removal of duplicated reads. We trimmed the raw reads to clip Illumina adapters, cut off low-quality bases at both ends, and discarded reads less than 60 bp in length after trimming using the Trimmomatic (version 0.33, parameters: ILLUMINACLIP : TruSeq3-PE-2.fa:2:30:10:8:true LEADING:20 TRAILING:20 MINLEN:60). We aligned quality-filtered reads to the human reference genome (hg19) using bowtie2 with default parameters (version 2.3.2) and BMTagger (version 3.101). We kept only reads of which both paired ends failed to align in either tool. The average rates of host DNA contamination were 0.12% for fecal samples. As a final QC step, we removed duplicate reads using PRINSEQ-lite (version 0.20.4, parameters: -derep 1). We excluded one MS sample due to an extremely small number of QC passed reads and another MS sample due to the outlier in principal component analysis (PCA) of both phylogenetic data and gene abundance data (described below).

### Taxonomic Annotation of Metagenome and Abundance Quantification

To improve both the efficiency and accuracy of taxonomic assignment, we selected the reference metagenomes of the Japanese population constructed by Nishijima *et al*. (Nishijima et al., 2016); 6,139 genomes from the National Center for Biotechnology Information and 10 genomes from in-house complete genome data constructed at Osaka University. Furthermore, we added newly reported genomes from the cultivated human gut bacteria projects (Almeida et al., 2019; Forster et al., 2019; Zou et al., 2019). After filtration to the genomes annotated to the species with more than 50 reference genomes, the taxonomic reference genome dataset consisted of 7,881 genomes. The filtered paired-end reads were aligned to the reference genome datasets using bowtie2 with default parameters (version 2.3.2). The average mapping rate was 88%. As for multiple-mapped reads, only the best possible alignment was selected by the alignment scores. The number of reads that mapped to each genome was divided by the length of the genome. The value of each genome was summed up by each sample, and the relative abundance of each clade was calculated at six levels (L2: phylum, L3: class, L4: order, L5: family, L6: genus, L7: species). For removing batch effects indicative of contaminants, we excluded clades that had been detected in neither of our previous metagenome cohorts (31 samples with average 29 Gb per sample and 96 samples with average 8.1 Gb, respectively) (Kishikawa et al., 2020a). At last we detected outlier samples by PCA.

### Functional Annotation and Abundance Calculation

*De novo* assembly of the filtered paired-end reads into contigs was conducted using MEGAHIT (version 1.1.2, parameters: –min-contig-len 135). We predicted open reading frames (ORFs) on the contigs with the *ab initio* gene finder MetageneMark (version 3.38, parameters: -a -k -f G). Next, we annotated the ORF catalog with two protein databases, UniRef90 (Suzek et al., 2015) and Kyoto Encyclopedia of Genes and Genomes (KEGG; (Kanehisa and Goto, 2000). For the UniRef90 database, we selected prokaryotic, viral, and fungal data. For KEGG genes, we utilized a database of prokaryote KEGG genes and MGENES, a database of KEGG genes from metagenome samples annotated based on orthology, with a bit score >60. We aligned putative amino acid sequences translated from the ORF catalog against both databases with DIAMOND using BLASTP (v0.9.4.105, parameters: f 6 -b 15.0–k 1 -e 1e-6 –subject-cover 50). We identified 2,058,642 UniRef proteins and 1,248,480 KEGG genes. For quantification of ORF abundance, we mapped the filtered paired-end reads to the assembled contigs using bowtie2 with default parameters (version 2.3.2). To avoid the bias of the gene size, the ORF abundance was defined as the depth of each ORF’s region of the ORF catalog according to the mapping result. As well as phylogenetic data, we excluded genes that had been detected in neither of our previous metagenome cohorts (Kishikawa et al., 2020a) and detected outlier samples by PCA. As mentioned above, one MS sample was excluded because it was the outlier of both phylogenetic data and gene abundance data.

### Case-Control Association Test for Phylogenetic Data

We normalized the relative abundance profiles using the Box-Cox transformation function in the car R package (version 3.0.2), including log transformation. We removed clades detected (i) in less than 20% of the samples, (ii) in no sample in either cases or controls, or (iii) with an average relative abundance of less than 0.001% of total abundance. After selection, we assessed 712 clades (10 phyla, 20 classes, 31 orders, 63 families, 166 genera, and 422 species). Case-control association tests were performed separately for each clade using the generalized linear model function in the R package glm2 (version 1.2.1). We adopted sex, age, and the top three principal components as covariates.

### Case-Control Association Test for Gene Abundance Data

We converted each ORF abundance to annotated gene abundance for both UniRef90 protein and KEGG gene databases. We performed two steps of normalization. First, we adjusted the gene abundance by the sum of ORF abundance for each sample in order to correct the bias of the amount of sequence reads for each sample. Next, we applied a rank-based inverse normal transformation in order to correct the heterogeneity of each gene’s abundance and distribution. We removed genes detected (i) in less than 20% of the samples or (ii) in no sample in either cases or controls. After gene selection, we assessed 219,715 genes annotated by the UniRef90 database and 222,606 genes annotated by the KEGG gene database. Case-control association tests were performed using the generalized linear model function in the R package glm2 (version 1.2.1). We adopted sex and age as covariates.

### Metagenome Molecular Pathway Analysis

We performed gene set enrichment analysis using the R package clusterProfiler (version 3.8.1). Gene sets which contained over 30,000 genes or under 50 genes were excluded from the enrichment analysis. For case-control pathway association tests, genes annotated by the UniRef90 database were ranked based on their effect sizes of case-control gene association tests. The UniRef90 gene sets were composed according to Gene Ontology (GO) (Harris et al., 2004). Genes annotated by the KEGG gene database were ranked in the same way. The KEGG gene sets were defined according to the KEGG pathway.

### Comparison of Gene Ontology Enrichment Analysis Results Between Multiple Sclerosis Metagenome and Host Genome-Wide Association Study

We assessed whether there were shared biological pathways between the gut metagenome and the human germline genome; we compared the GO enrichment data of the metagenome with that of the host GWAS in MS. For the host GWAS in MS, we obtained summary statistics from MS GWAS in the European population (*n* = 41,505) (International Multiple Sclerosis Genetics Consortium, 2019). We used Pascal with the summary statistics in order to determine GO enrichment of the germline in MS. We compared the *P*-values of GO shared between the GWAS data and metagenome data. We evaluated the overlap of the GO enrichment, by classifying the pathways based on the significance threshold of *P* < 0.05 or *P* ≥ 0.05 and using Fisher’s exact test.

### Empirical Estimation of Metagenome-Wide Significance Threshold

We empirically estimated the statistical significance threshold separately for phylogenetic and gene case-control analyses, performing a phenotype permutation procedure (Kanai et al., 2016). We randomly simulated case-control phenotypes (×50,000 iterations) and calculated empirical null distributions of the minimum *P*-values (= *P*_min_) in each iteration. We defined an empirical Bonferroni significance threshold, -log_10_(*P*_sig_), as the 95th percentile of −log_10_(*P*_min_) at a significance level of 0.05. We calculated -log_10_(*P*_sig_) using the Harrell-Davis distribution-free quantile estimator (Harrell and Davis, 1982) and calculated a 95% confidence interval for -log_10_(*P*_sig_) by a bootstrapping method in the R package Hmisc (version 4.1.1). To estimate the null distribution of the test statistics, we applied the same process used for minimum *P*-values to all the ranked *P*-values. We defined an empirical false discovery rate (FDR) threshold of 0.1 as the 90th percentile of -log_10_ *P*-values of each rank at a significance level of 0.1.

### Multiple Sclerosis Case-Control Difference Between Alpha-Diversity and Beta-Diversity of the Metagenome

For calculating diversities, all samples were down-sampled at the same number of reads (*n* = 3,000,000). Alpha-diversity (within-sample diversity) was calculated based on gene abundance and six levels of phylogenetic relative abundance (L2–L7) for each sample according to the Shannon index. Statistical comparisons of Shannon index between MS cases and controls were assessed by Student’s t-test. To quantify beta-diversity, non-metric multidimensional scaling on the Bray-Curtis dissimilarity was performed. For evaluating case-control differences in the dissimilarity, we performed permutational multivariate analysis of variance (PERMANOVA) (McArdle and Anderson, 2001) with 100,000 permutations using the R package vegan (version 2.5.4).

## Results

### Shotgun Sequencing of MS Microbiome in the Japanese Population

We performed whole-genome shotgun sequencing of a total of 103 fecal DNA samples (26 individuals with MS and 77 healthy controls of Japanese ancestry; **Supplementary Table 1**), which passed stringent QC filters for the sequence reads and samples as described elsewhere (Kishikawa et al., 2020a). High-throughput whole-genome shotgun sequencing achieved relatively high read amounts per sample (average 7.3Gb), enabling robust implementation of a series of MWAS analyses.

### Identification of Multiple Clades With Multiple Sclerosis Case-Control Discrepancy

**Figure 1:**
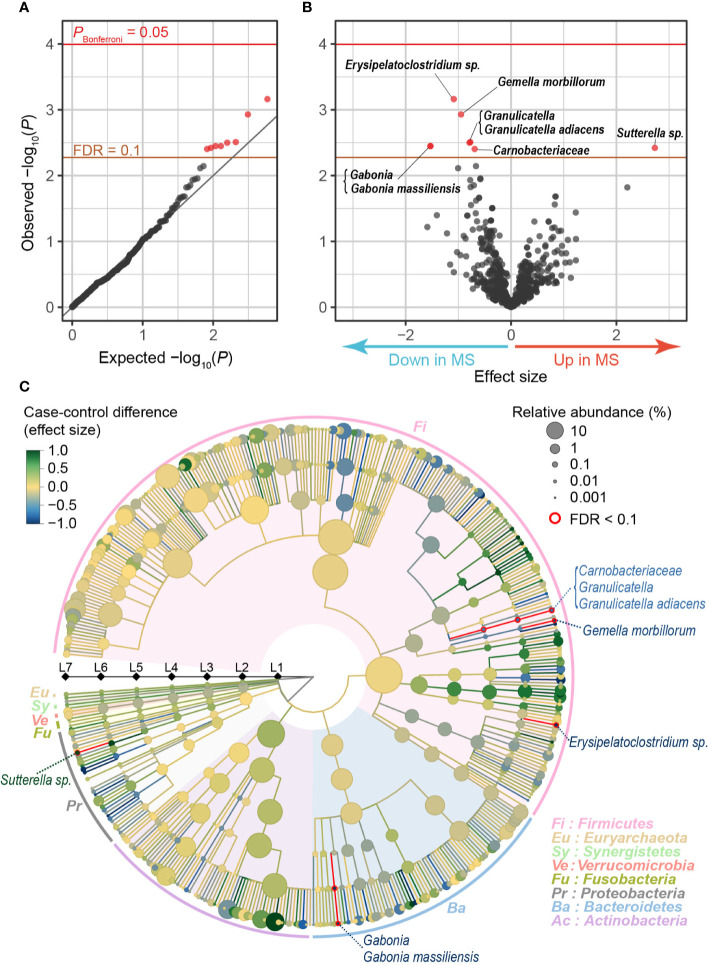
MWAS results of MS case-control phylogenetic association tests. **(A)** A quantile-quantile plot of the MWAS *P*-values of the clades. The *x*-axis indicates empirically estimated median -log_10_ *P*-values. The *y*-axis indicates observed -log_10_ *P*-values. The diagonal gray line represents *y* = *x*, which corresponds to the null hypothesis. The horizontal red line indicates the empirical Bonferroni-corrected threshold (*α* = 0.05), and the brown line indicates the empirically estimated (FDR-*q* = 0.1). Clades with FDR-*q* < 0.1 are plotted as red dots, and other clades as black dots. **(B)** A volcano plot. The *x*-axis indicates effect sizes of generalized linear model. The *y*-axis, horizontal lines, and dot colors are the same as in panel **(A)**. **(C)** Phylogenetic tree. Levels L2–L7 are from the inside layer to the outside layer. The size and color of dots represent relative abundance and effect sizes, respectively. The five clades with suggestive case-control associations (FDR-*q* < 0.1) are outlined in red. FDR, false discovery rate; MWAS, metagenome-wide association study; MS, multiple sclerosis.

After stringent QC for sequence reads and samples (**Supplementary Figure 1**), our MWAS assessed a total of 712 clades (10 phyla [L2], 20 classes [L3], 31 orders [L4], 63 families [L5], 166 genera [L6], and 422 species [L7]). Case-control phylogenetic association tests using a generalized linear regression model identified eight clades which conferred case-control discrepancy in their composition levels (empirically estimated FDR-*q* < 0.1; **Table 1**, **Figure 1A**). Of these, one exhibited increased abundances in the MS samples than in the controls (*Sutterella* sp.), whereas seven exhibited decreased abundances (*Erysipelatoclostridium* sp., *Gemella morbillorum*, *Granulicatella*, *Granulicatella adiacens*, *Gabonia*, *Gabonia massiliensis*, and *Carnobacteriaceae*; **Figure 1B**). Our analysis adjusted the confounding effects of sex and age by incorporating them as covariates. Furthermore, we confirmed that the results were independent of age and sex by conducting the stratified analyses (**Supplementary Table 2**). *Granulicatella adiacens* (L7), the genus *Granulicatella* (L6), and the family *Carnobacteriaceae* (L5), which were a series of the identical strain, showed significant correlations with age in MS samples, but not significant in control samples or all samples. We confirmed that the abundances of the clades in MS were less than those in controls across most generations (**Supplementary Figure 2**). To assess the correlation between the disease severity and the eight clade abundances, we divided the MS patients into two groups of severe MS (Expanded Disability Status Scale [EDSS] ≥ 4.5; *n* = 8) and mild MS (EDSS < 4.5; *n* = 18). We found no significant differences between the two groups, whereas severe MS exhibited 15.9 times more mean abundance of *Sutterella* sp. than that of mild MS (**Supplementary Table 3**).

**Table 1:** Clades in gut microbiome with MS case-control discrepancy.

Microbe	Level	Effect size	*P*-value
*Erysipelatoclostridium* sp.	Species (L7)	−1.09	6.9 × 10^−4^
*Gemella morbillorum*	Species (L7)	−0.95	0.0012
*Granulicatella*	Genus (L6)	−0.77	0.0031
*Granulicatella adiacens*	Species (L7)	−0.79	0.0032
*Gabonia*	Genus (L6)	−1.53	0.0036
*Gabonia massiliensis*	Species (L7)	−1.53	0.0036
*Sutterella* sp.	Species (L7)	2.73	0.0038
*Carnobacteriaceae*	Family (L5)	−0.69	0.0039

MS, multiple sclerosis.

As for the characteristics of the clades with case-control discrepancies, the genus *Erysipelatoclostridium* is mostly clostridium cluster XVIII reported to produce acetate, one of short-chain fatty acid (SCFA) (Narushima et al., 2014). SCFA plays a critical role in suppressing inflammation by inducing Tregs (Smith et al., 2013). SCFA was reported to ameliorate EAE (Haghikia et al., 2015) and modify blood-brain barrier permeability (Braniste et al., 2014). *Granulicatella adiacens*, the genus *Granulicatella*, and the family *Carnobacteriaceae* produce lactic acid (Siqueira and Rôças, 2006). An oral administration of the lactic acid bacterium ameliorated clinical EAE (Takata et al., 2011). *Gemella* was reported to reduce IL-12 levels in oral infections in mice (Ribeiro Sobrinho et al., 2002), while the role of *Gemella* in gut microbiome is unclear. IL-12 was one of the MS-associated genes found in MS GWAS (International Multiple Sclerosis Genetics Consortium, 2019). Thus, we detected the novel MS-associated clades related to the immune system. The clades could influence the pathology of MS. On the other hand, *Gabonia massiliensis* is a new species officially registered in 2015 (Lee et al., 2011; Mourembou et al., 2015). There have been few reports of the correlation of the clade and autoimmune diseases so far.

As for the previously reported MS-related clades of gut microbiome (Jangi et al., 2016; Cekanaviciute et al., 2017), we observed nominally significant associations in *Parabacteroides distasonis* (*P* = 0.01; **Supplementary Table 4**), providing rigorous evidence in our MWAS framework.

As illustrated in a phylogenetic tree indicating the case-control association results of multi-layered taxonomic levels (**Figure 1C**), five of the eight clades with case-control discrepancy belonged to species (L7, the most specific level). Since it was difficult to detect the species-level clades using classical 16S rRNA sequencing, our results clearly demonstrated the value of metagenome shotgun sequencing and the MWAS approach to identify disease-associated microbiome taxa. Each of the five species with case-control discrepancy belonged to different genera (L6) or families (L5), which was comparable to the previous reports that the majority of the disease-associated taxa belonged to relatively limited variety of genera or families (e.g., *Prevotella* families in rheumatoid arthritis [Maeda et al., 2016; Kishikawa et al., 2020a]). Our results should empirically propose polytaxonomic architecture of MS microbiome rather than monotaxonomic one, which represents contribution of relatively wider ranges of taxa with moderate effects.

#### High Abundance of an ABC Transporter-Related Gene in Multiple Sclerosis Metagenome

**Figure 2:**
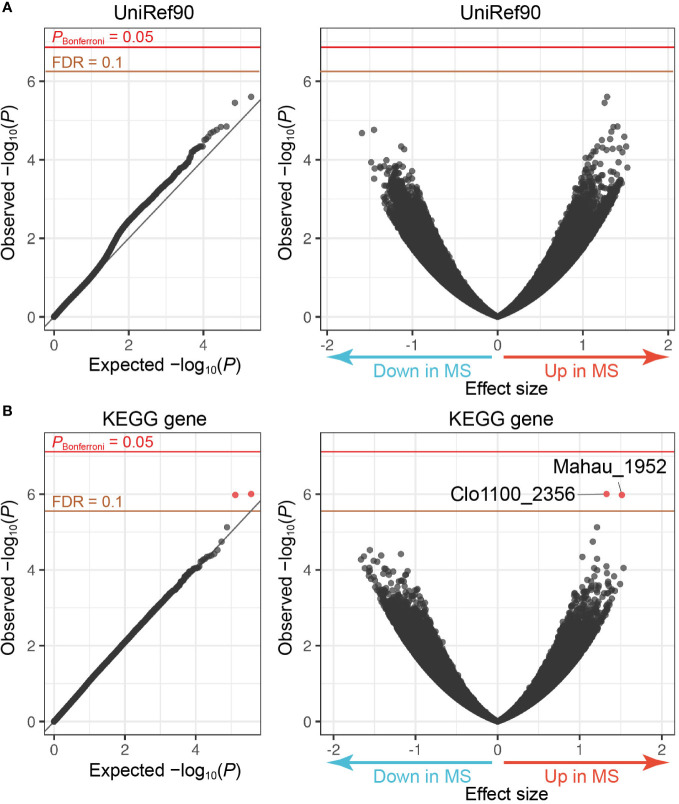
MWAS results of MS case-control gene association tests. **(A)** A quantile-quantile plot (left) and a volcano plot (right) of the MWAS *P*-values of genes based on the UniRef90 protein database. **(B)** A quantile-quantile plot (left) and a volcano plot (right) of genes based on the KEGG gene database. In the quantile-quantile plots, the *x*-axis indicates empirically estimated median -log_10_ *P*-values. In the volcano plot, the *x*-axis indicates beta of generalized linear model as effect sizes. The *y*-axis in both plots indicates observed -log_10_ *P*-values. The diagonal gray line represents *y* = *x*, which corresponds to the null hypothesis. The horizontal red line indicates the empirical Bonferroni-corrected threshold (*α* = 0.05), and the brown line indicates the empirically estimated (FDR-*q* = 0.1). Genes with FDR-*q* < 0.1 are plotted as red dots, and other genes as black dots.

The MWAS framework can quantitatively assess case-control discrepancy of gene abundances in metagenome, which deserves discovery of novel therapeutic targets. To this end, we performed the following procedures: (i) *de novo* assembly, (ii) prediction of ORFs, and (iii) calculating the ORF abundances by mapping the reads to the assembled contigs. After QC for the genes, we obtained 219,715 and 222,606 genes annotated by the UniRef90 and KEGG databases, respectively. Case-control gene association tests utilizing a generalized linear regression model found that two genes registered at KEGG increased in the MS samples compared to the controls (FDR-*q* < 0.1, Clo1100_2356 and Mahau_1952; **Figure 2** and **Table 2**). Clo1100_2356 is categorized in glycerol-3-phosphate dehydrogenase, and Mahau_1952 is defined as ABC transporter related protein. It has been suggested that ABC transporter of microbiome affects the etiology of neuromyelitis optica (NMO), an MS-related disease. T cells specific for aquaporin 4, the major antigen of NMO, cross-react with the homologous ABC transporter peptide of *Clostridium perfringens*, suggesting that molecular mimicry against microbial antigens could induce the autoimmune response in NMO (Varrin-Doyer et al., 2012). Although the specific antibody of MS has never been detected, the increased abundance of ABC transporter related gene in MS metagenome implies a similar immune mechanism in MS.

**Table 2:** Metagenome genes with MS case-control discrepancy.

KEGG gene	Effect size	*P*-value	Definition	KEGG Orthology
Clo1100_2356	1.33	9.9 × 10^−7^	putative dehydrogenase	glpA, glpD; glycerol-3-phosphate dehydrogenase
Mahau_1952	1.54	1.1 × 10^−6^	ABC transporter related protein	ABC-2.A; ABC-2 type transport system ATP-binding protein

KEGG, Kyoto Encyclopedia of Genes and Genomes; MS, multiple sclerosis.

We evaluated the effects of sex and age on the abundances of these two genes, founding no significant effects (**Supplementary Table 2**). Although *P*-value of sex differences of Mahau_1952 in MS samples was less than 0.05, the direction of the increase or decrease was opposite to the result of case-control association test; the abundances in males were more than those in females. There existed no significant effects of EDSS as well (**Supplementary Table 3**).

#### Alteration of Pathways Related to Lipopolysaccharide in Multiple Sclerosis

**Figure 3:**
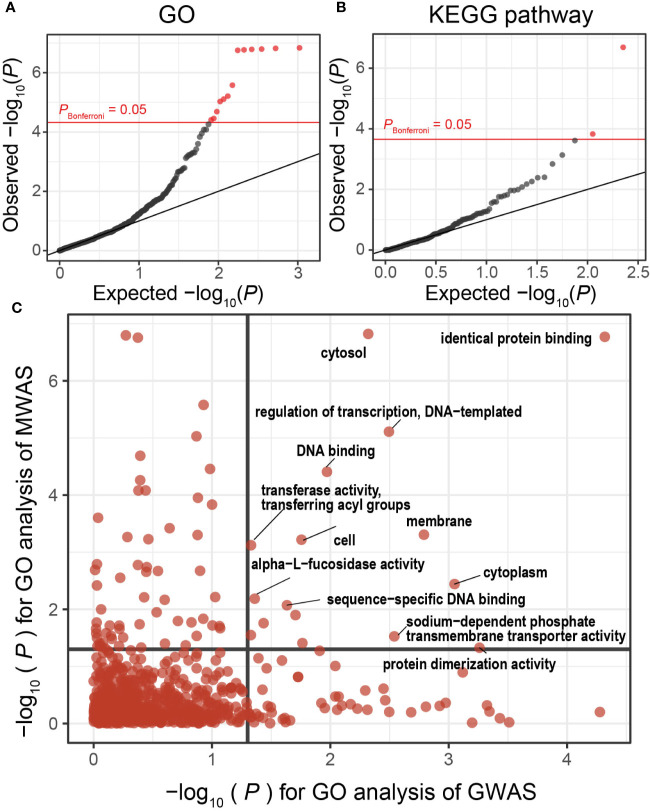
MWAS results of MS case-control pathway association tests. **(A)** A quantile-quantile plot of the MWAS *P*-values of enrichment analyses based on GO terms. GO terms with *P*-values less than Bonferroni thresholds are plotted as red dots, and the other clades as black dots. **(B)** A quantile-quantile plot of the MWAS *P*-values of enrichment analyses based on KEGG pathways. **(C)** Comparison of *P*-values of GO enrichment analyses between the MS MWAS and GWAS data. The *x*-axis indicates the *P*-values of the GWAS. The *y*-axis indicates the *P*-values of the MWAS. The horizontal and vertical black lines indicate *P*-value of 0.05. The overlap of the GO enrichment was evaluated by classifying the GO terms based on the significance threshold of *P* < 0.05 or *P* ≥ 0.05 and using Fisher’s exact test. GO, Gene Ontology; GWAS, genome-wide association study; KEGG, Kyoto Encyclopedia of Genes and Genomes; MWAS, metagenome-wide association study; MS, Multiple sclerosis.

We then performed gene set enrichment analysis to conduct case-control pathway association tests using the results of the gene analysis of our MWAS. We found significant associations for 13 GO terms and 2 KEGG pathways that satisfied the Bonferroni’s correction (**Figures 3A, B**, and **Table 3**). One of the GO terms with significant enrichments was cell outer membrane (*P* = 1.5 × 10^−7^, GO:0009279). Major component of the outer membrane of gram-negative bacteria was lipopolysaccharides (LPS), which prompts activation of cell and secretion of inflammatory cytokines by engaging Toll-like receptor (Stevens et al., 2015). As for MS, LPS has been reported to induce and worsen experimental autoimmune encephalomyelitis (Nogai et al., 2005). We also found that KEGG pathway of lipopolysaccharide biosynthesis (*P* = 2.1 × 10^−7^, ko00540) was significantly altered. Although the involvement of LPS in MS is already known, this study is first to robustly demonstrate the involvement using functional analysis of shotgun sequencing.

**Table 3:** Pathways with case-control discrepancy in MS MWAS.

GO term	Set size	*P*-value	Name
GO:0009279	572	1.5 × 10^−7^	cell outer membrane
GO:0005829	408	1.5 × 10^−7^	cytosol
GO:0015562	228	1.6 × 10^−7^	efflux transmembrane transporter activity
GO:0030288	176	1.6 × 10^−7^	outer membrane-bounded periplasmic space
GO:0042802	102	1.7 × 10^−7^	identical protein binding
GO:0006974	54	1.8 × 10^−7^	cellular response to DNA damage stimulus
GO:0000272	85	2.6 × 10^−6^	polysaccharide catabolic process
GO:0042597	190	6.2 × 10^−6^	periplasmic space
GO:0006355	4053	7.8 × 10^−6^	regulation of transcription, DNA-templated
GO:0005886	9545	9.4 × 10^−6^	plasma membrane
GO:0004853	69	2.1 × 10^−5^	uroporphyrinogen decarboxylase activity
GO:0008422	177	3.5 × 10^−5^	beta-glucosidase activity
GO:0003677	20242	3.9 × 10^−5^	DNA binding
**KEGG pathway**	**Set size**	***P*-value**	**Name**
ko00540	318	2.1 × 10^−7^	Lipopolysaccharide biosynthesis
ko00790	794	1.5 × 10^−4^	Folate biosynthesis

GO, gene ontology; MS, multiple sclerosis; MWAS, metagenome-wide association study.

#### Evidence of Shared Molecular Pathways Between the Metagenome and Host Genome in Multiple Sclerosis

Disentanglement of interaction between host genomics and metagenome is a key concept towards elucidation of disease etiology. While human host genes and gene products of microbiome were categorized as different omics layers, one can directly connect them though *in silico* trans-omics connection by projection to the molecular pathways (Kishikawa et al., 2020a). To this end, we evaluated whether disease-associated molecular pathways represented as the GO terms were shared between the gut metagenome and the host germline genome. In addition to the GO enrichments of the MWAS mentioned above, we estimated GO enrichments in the association signals observed in the previously conducted MS GWAS data (*n* = 41,505) (International Multiple Sclerosis Genetics Consortium, 2019). We quantified MWAS-GWAS interaction by comparing the *P*-values of the GO terms shared between the MS MWAS data and the MS GWAS data (**Figure 3C**). Multiple GO terms showed significant enrichments between the metagenomes and host genomes of MS (*P*_GO_ < 0.05 in both). Of these, the identical protein binding term demonstrated notably significant enrichments (*P*_GO_ = 1.7 × 10^−7^ and 4.8 × 10^−5^ in MWAS and GWAS, respectively), suggesting its prominent roles in MS pathophysiology. We observed a significant correlation between *P*-values of GO terms (*P*_Fisher_ = 1.7 × 10^−4^). This MWAS-GWAS interaction provided empirical evidence of biological link between the germline genome and metagenome in MS pathology.

#### No Apparent Discrepancies in Metagenome Diversity Between Multiple Sclerosis Cases and Controls

**Figure 4:**
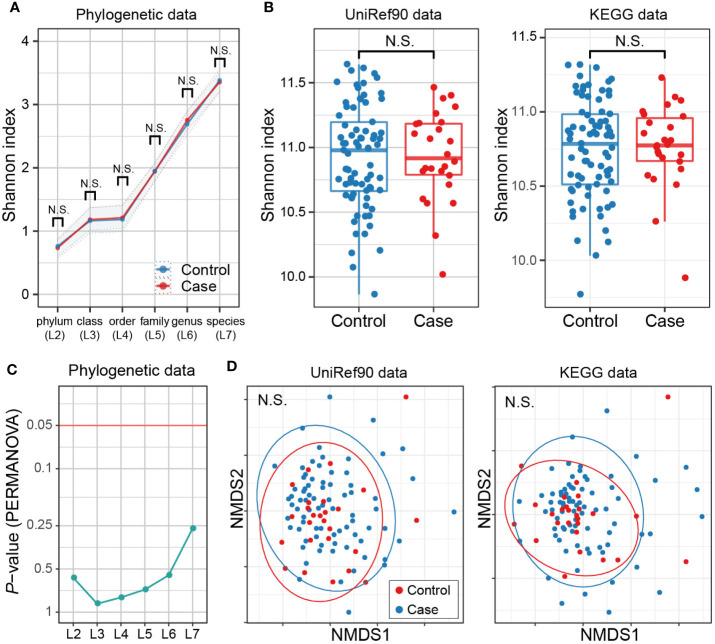
MS case-control comparison of microbial diversities. **(A)** Alpha diversities of the phylogenetic relative abundance data for six levels. Welch’s t-test of Shannon index between MS cases and controls showed no significant difference at any level. **(B)** Alpha diversities of the gene abundance data of the UniRef90 protein and KEGG gene databases. No significant case-control difference was found. **(C)** Beta diversities of phylogenetic relative abundance data at six levels. PERMANOVA based on Bray-Curtis dissimilarities found no significant differences among levels for either sequencing group with Bonferroni correction. **(D)** Beta diversities of the gene abundance of the UniRef90 protein database. No significant case-control difference was found. KEGG, Kyoto Encyclopedia of Genes and Genomes; NMDS, non-metric multidimensional scaling; PERMANOVA, permutational multivariate analysis of variance; MS, Multiple sclerosis.

There have been studies assessing whether taxonomic diversities of gut microbiome differ between MS cases and controls or not (Miyake et al., 2015; Chen et al., 2016; Jangi et al., 2016; Berer et al., 2017; Cekanaviciute et al., 2017), along with the discussions on how to locate dysbiosis in the pathogenicity of MS. We thus assessed alpha- and beta-diversity in both of the phylogenetic data (phylogenetic relative abundance of six levels [L2–L7]) and functional data (gene abundance based on the UniRef90 protein and KEGG gene databases). Not any levels of phylogenetic data showed significant case-control differences in alpha-diversity based on the Shannon index (*P* > 0.05; **Figure 4A**). The gene abundance data did not show significant differences either (*P* > 0.05; **Figure 4B**). Similarly, no significant differences in beta-diversity were found in any case-control comparisons (*P* > 0.05; **Figures 4C, D****)**. Overall, there exist no apparent discrepancies in metagenome diversity between MS cases and controls.

## Discussion

In this study, we conducted a comprehensive MWAS of MS in the Japanese population, by utilizing whole-genome shotgun sequencing. Our study identified the following novel features of the MS gut metagenome: (i) Eight clades, mostly related to immune systems, showed discrepancies in the case-control comparison. (ii) The abundances of two genes increased in the MS metagenome, including the ABC transporter related gene which could induce autoimmune response through molecular mimicry. (iii) Molecular pathways related to LPS prompting secretion of inflammatory cytokines were altered in the MS metagenome. (iv) Significant interaction of the pathways between the metagenome (MWAS) and the host genome (GWAS) was identified. (v) No apparent discrepancies in metagenome diversities were found between the MS cases and controls. Our study greatly exploited the benefits of shotgun sequencing, since these characteristics would be difficult to find by using the classical method of 16S rRNA.

Our study has an advantage in high resolution analysis focusing the species level (L7). Of the eight clades with MS case-control discrepancy in our study, the genus *Sutterella* has been reported to increase in the gut microbiome of MS patients under treatment compared to healthy controls, while decreasing in untreated MS patients (Jangi et al., 2016). In our study, there existed a higher relative abundance of *Sutterella* sp. in the MS patients under treatment than in the untreated MS patients (**Supplementary Table 3**). When stratifying the MS patients by both severity of MS and treatment status, it was suggested that they influenced the abundance of *Sutterella* sp. independently (**Supplementary Figure 3**). Thus, the increased level of *Sutterella* sp. in our study could be an effect of medication because most patients (90%) were under treatment. The other seven clades were not influenced by treatment status.

Our MWAS on genes and molecular pathways successfully found the novel functional aspects of the MS gut metagenome. In the taxonomic assignment of Clo1100_2356, The Clo1100_2356 sequences in our metagenome data were mainly linked to the taxonomic reference genomes of *Firmicutes bacterium* and *Ruminococcus* sp., while the source organism is registered as *Clostridium* sp. in the KEGG database. The Mahau_1952 sequences in our metagenome data were mainly linked to the taxonomic reference genomes of *Dorea formiciigenerans* and *Lachnospiraceae bacterium*. We found no direct association between our findings of phylogenetic analysis and gene analysis.

The studies focusing on the interaction between the metagenome and host genome in human complex diseases have become an interesting topic in the field of microbiome (Imhann et al., 2018; Asquith et al., 2019). Our study is the first to demonstrate the MWAS-GWAS interaction of the molecular pathways in the MS gut metagenome. Our results should warrant further studies to elucidate functional connection between MS metagenome and host genome.

Ma et al. performed a comprehensive analysis of the microbial diversity in microbiome-associated diseases (not including MS) and indicated that there were no significant differences in most diseases relative to controls (Ma et al., 2019). Our results supported this finding for MS in the aspects of both taxonomic and functional gene diversity. Thus, MS gut microbiome is characterized by combination of specific disease-associated clades, genes, and molecular pathways rather than overall dysbiosis.

In this study, age and sex between case and control were not completely matched. However, we corrected for their bias by analyzing with incorporation of sex and age as covariates. We also confirmed that age and sex did not significantly affect the genes and clades with MS case-control discrepancies. Furthermore, Odamaki et al. examined changes in the gut microbiome composition of the Japanese population with age in a wide range of age groups from newborn to centenarian (Odamaki et al., 2016). They demonstrated that the microbiome composition remained relatively stable between the 20s and 60s, the relevant age group in our study. Thus, we may detect fewer findings than expected by adjusting confounding effects, but the results of this study are robust regardless of age and sex.

## Conclusions

In conclusion, our shotgun sequencing-based comprehensive MWAS revealed novel characteristics of the MS gut microbiome and the interaction between the gut metagenome and host genome. Our study will provide useful resources for future functional investigations to further elucidate details of the microbiome’s role in MS etiology.

## Data Availability Statement

The whole-genome shotgun sequencing data are deposited in National Bioscience Database Center (NBDC) Human Database (http://humandbs. biosciencedbc. jp/) with the accession number of hum0197. The data are available upon reasonable request.

## Ethics Statement

The studies involving human participants were reviewed and approved by the ethical committees of Osaka University. The patients/participants provided their written informed consent to participate in this study.

## Author Contributions

TK and YO designed the study, conducted the data analysis, and wrote the manuscript. TK, DM, YMat, TN, YMae, and SN conducted the experiments. TK, KO, AH, MK, KS, KY, TM, SN, HI, and TO collected the samples. HI, HM, TO, and YO supervised the study. All authors contributed to the article and approved the submitted version.

## Funding

This research was supported by the Japan Society for the Promotion of Science (JSPS) KAKENHI (19H01021 and 20K21834), the Japan Agency for Medical Research and Development (AMED; JP20ek0109413, JP20km0405211, JP20ek0410075, JP20gm4010006, and JP20km0405217), Takeda Science Foundation, Bioinformatics Initiative of Osaka University Graduate School of Medicine, Grant Program for Next Generation Principal Investigators at Immunology Frontier Research Center (WPI-IFReC), Osaka University.

## Conflict of Interest

The authors declare that the research was conducted in the absence of any commercial or financial relationships that could be construed as a potential conflict of interest.

## Abbreviations

EAE, experimental autoimmune encephalomyelitis; EDSS, Expanded Disability Status Scale; FDR, false discovery rate; GO, Gene Ontology; GSEA, gene set enrichment analysis; GWAS, genome-wide association study; KEGG, Kyoto Encyclopedia of Genes and Genomes; LPS, lipopolysaccharides; MWAS, metagenome-wide association study; NGS, next generation sequencing; NMO, neuromyelitis optica; ORFs, open reading frames; PCA, principal component analysis; PERMANOVA, permutational multivariate analysis of variance; QC, Quality control; RRMS, relapsing-remitting multiple sclerosis; rRNA, ribosomal RNA; SCFA, short-chain fatty acid.
