# Supplementary material for: A Metagenome-Wide Association Study of Gut Microbiome in Patients With Multiple Sclerosis Revealed Novel Disease Pathology
Source: Front Cell Infect Microbiol. 2020 Dec 11;10:585973. doi: 10.3389/fcimb.2020.585973 (PMC7759502; doi:10.3389/fcimb.2020.585973)
Supplement: Supplementary file 1 [file DataSheet_1.docx]

***Supplementary Material***

**A metagenome-wide association study of gut microbiome in multiple sclerosis revealed novel pathology.**

**Supplementary Material Contents**

The Supplementary Material comprises two supplementary Figures, three supplementary Tables, and URLs of analysis tools.

**Supplementary Figures**

Supplementary Figure 1. Bioinformatic pipelines for the metagenome-wide association study.

Supplementary Figure 2. Correlation of ages and abundances of genus *Granulicatella.*

Supplementary Figure 3. Boxplots of relative abundances of *Sutterella sp.* in the multiple sclerosis patients stratified by severity and treatment states.

**Supplementary Tables**

Supplementary Table 1. Characteristics of the study population.

Supplementary Table 2. Effects of sex and age in the abundances of the clades and gene with multiple sclerosis case-control discrepancies.

Supplementary Table 3. Effects of severity in the abundances of the clades and genes with MS case-control discrepancies.

Supplementary Table 4. Major clades previously reported for association with multiple sclerosis.

Supplementary Table 5. Effects of treatment status in the abundances of the clades and genes with MS case-control discrepancies.

**URLs**

**
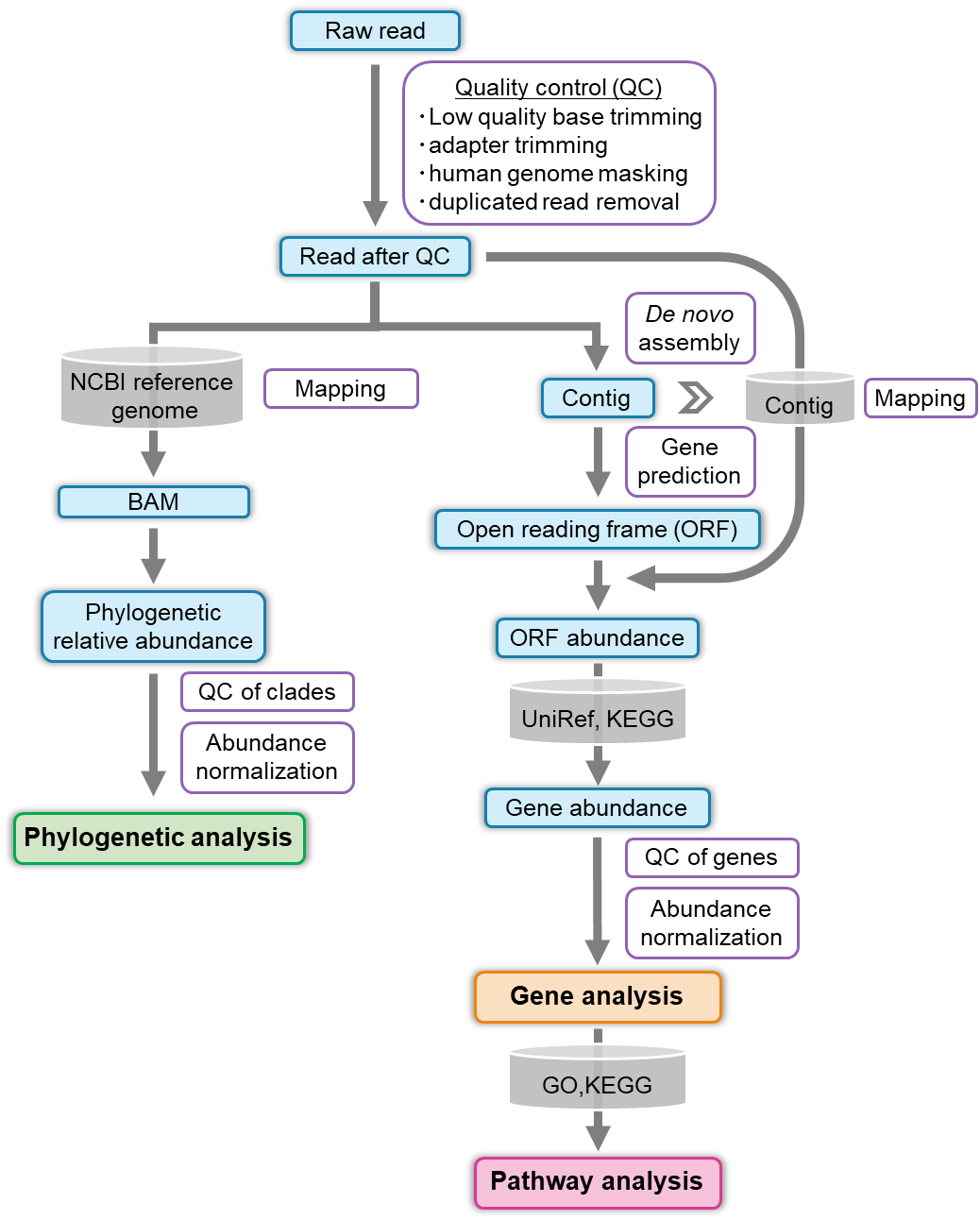
Supplementary Figure 1. Bioinformatic pipelines for the metagenome-wide association study.** Whole-genome shotgun sequencing reads of the gut microbiome were processed following this pipeline. It consisted of three major bioinformatic analytic techniques (phylogenetic analysis, functional gene analysis, and pathway analysis).


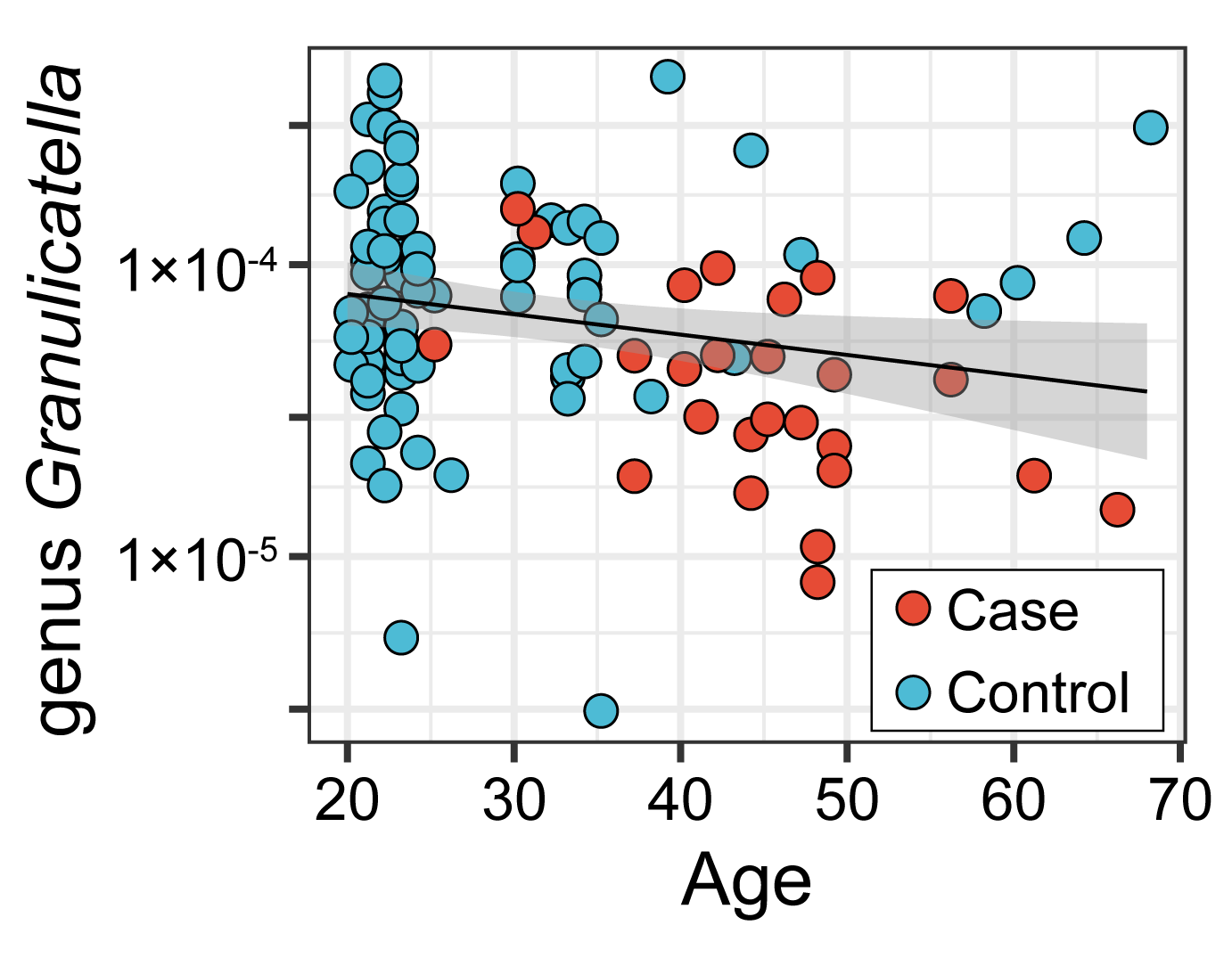


**Supplementary Figure 2. Correlation of ages and abundances of genus *Granulicatella*.**

The *y*-axis indicates relative abundances of *genus Granulicatella* in logarithmic scale.


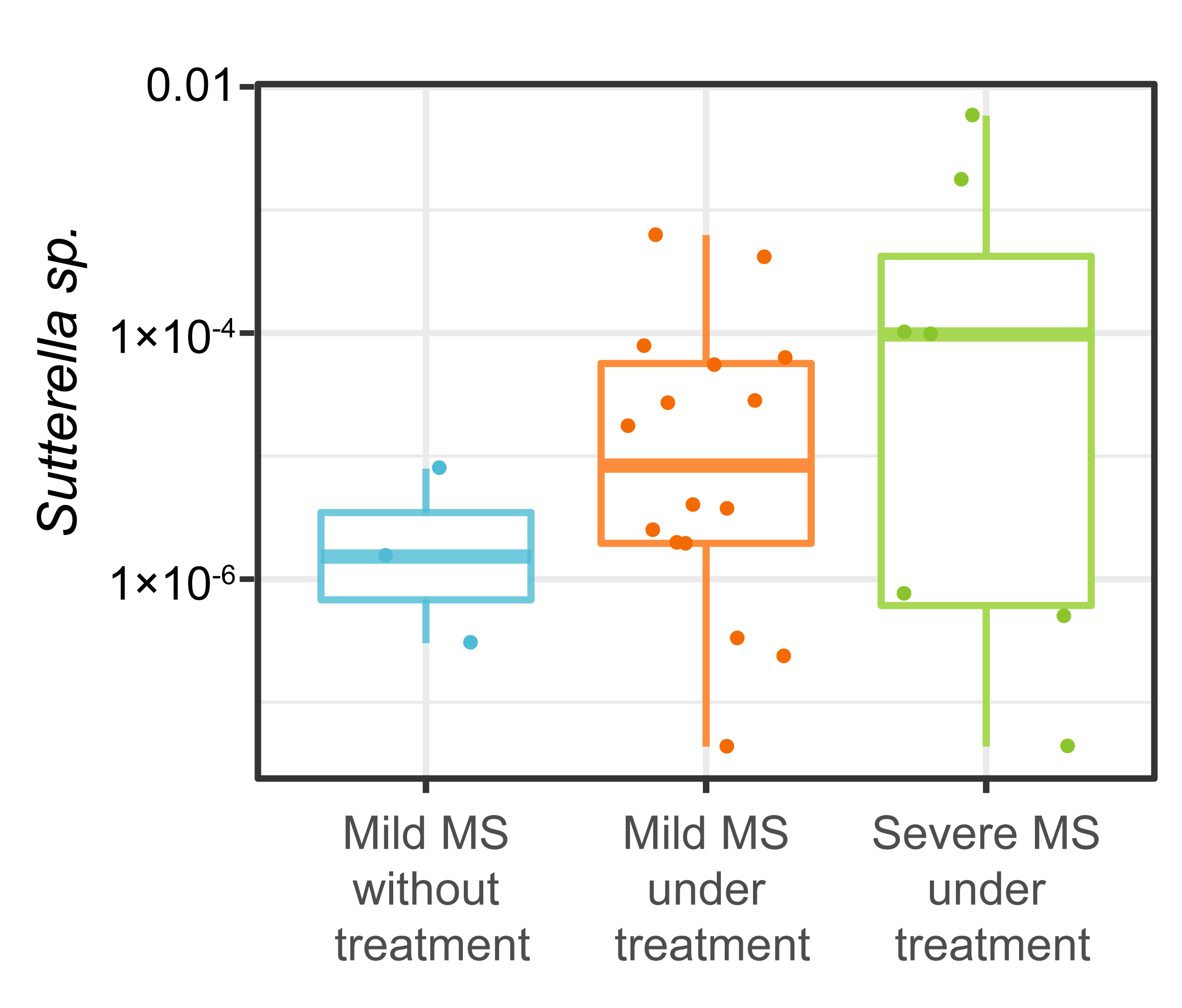


**Supplementary Figure 3. Boxplots of relative abundances of *Sutterella sp.* in the multiple sclerosis patients stratified by severity and treatment states.**

EDSS < 4.5 was defined as mild MS, while EDSS ≥ 4.5 was defined as severe MS. The *y*-axis indicates relative abundances of *Sutterella sp.* in logarithmic scale. The lower and upper hinges of the boxes indicate the first and third quartiles. The horizontal lines within the boxes indicate median levels.

**Supplementary Table 1. Characteristics of the study population.**

|  | Multiple sclerosis (*n* = 26) | Control (*n* = 77) |
| --- | --- | --- |
| Age, years, mean (range) | 44.8 (25 – 66) | 28.1 (20 – 68) |
| Female | 22 (85%) | 26 (33%) |
| Disease duration, years, mean (range) | 13 (0 – 41) |  |
| Immunomodulatory drug exposure  non-treatment  glatiramer acetate  dimethyl fumarate  fingolimod  interferon | 3 (12%)  5 (19%)  7 (27%)  7 (27%)  4 (15%) |  |
| Corticosteroids | 6 (23%) |  |
| EDSS ≥ 4.5 | 7 (27%) |  |
| EDSS, Expanded Disability Status Scale | | |

**Supplementary Table 2. Effects of sex and age in the abundances of the clades and gene with MS case-control discrepancies.**

| **Microbe** | ***P*-value of sex differences** | | ***P*-value of correlation with age** | |
| --- | --- | --- | --- | --- |
|  | MS | Control | MS | Control |
| *Erysipelatoclostridium sp.* | 0.25 | 0.83 | 0.67 | 0.85 |
| *Gemella morbillorum* | 0.28 | 0.45 | 0.65 | 0.71 |
| *Granulicatella* | 0.81 | 0.35 | **0.0013** | 0.39 |
| *Granulicatella adiacens* | 0.81 | 0.19 | **0.0015** | 0.52 |
| *Gabonia* | 0.35 | 0.49 | 0.61 | 0.84 |
| *Gabonia massiliensis* | 0.35 | 0.49 | 0.61 | 0.84 |
| *Sutterella sp.* | 0.81 | 0.19 | 0.98 | 0.84 |
| *Carnobacteriaceae* | 0.71 | 0.33 | **0.005** | 0.42 |
| **Gene** | ***P*-value of sex differences** | | ***P*-value of correlation with age** | |
|  | MS | Control | MS | Control |
| Clo1100_2356 | 0.5 | 0.97 | 0.23 | 0.88 |
| Mahau_1952 | **0.012** | 0.13 | 0.11 | 0.15 |

**Supplementary Table 3. Effects of severity in the abundances of the clades and genes with MS case-control discrepancies.**

| **Clade** | **Fold-change*^1^** | ***P*-value*^2^** |
| --- | --- | --- |
| *Erysipelatoclostridium sp.* | 1.06 | 0.73 |
| *Gemella morbillorum* | 0.94 | 0.43 |
| *Granulicatella* | 0.88 | 0.78 |
| *Granulicatella adiacens* | 0.91 | 0.78 |
| *Gabonia* | 0.88 | 0.60 |
| *Gabonia massiliensis* | 0.88 | 0.60 |
| *Sutterella sp.* | 15.9 | 0.44 |
| *Carnobacteriaceae* | 0.91 | 0.91 |
| **Gene** |  |  |
| Clo1100_2356 | 1.72 | 0.38 |
| Mahau_1952 | 0.52 | 0.60 |
| *^1^ Under treatment / Non treatment  *^2^ Wilcoxon rank sum test | | |

**Supplementary Table 4. Major clades previously reported for association with MS.**

| **Microbe** | **Abundance in MS**  **of previous studies** | **Abundance in MS**  **of this study** | **Log FC**  **of this study** | ***P*-value**  **of this study** |
| --- | --- | --- | --- | --- |
| *Acinetobacter calcoaceticus^1^* | Up | deficient data | deficient | deficient |
| *Akkermansia^1-3^* | Up | Up | 0.63 | 0.78 |
| *Butyricimonas^2,4^* | Down | Down | -0.36 | 0.29 |
| *Clostridium^5,6^* | Various | Down | 0.03 | 0.22 |
| *Methanobrevibacter^2,4,7^* | Up | Up | 6.12 | 0.65 |
| *Parabacteroides distasonis^1^* | Down | Down | -0.84 | 0.01 |
| *Prevotella^2,5^* | Down | Down | -1.66 | 0.49 |
| *Streptococcus thermophilus^5^* | Up | Down | -0.54 | 0.72 |
| *Sutterella^2,3,5^* | Down | Down | -0.60 | 0.21 |

1. Cekanaviciute, E., Yoo, B.B., Runia, T.F., Debelius, J.W., Singh, S., Nelson, C.A., Kanner, R., Bencosme, Y., Lee, Y.K., Hauser, S.L., Crabtree-Hartman, E., Sand, I.K., Gacias, M., Zhu, Y., Casaccia, P., Cree, B.a.C., Knight, R., Mazmanian, S.K., and Baranzini, S.E. (2017). Gut bacteria from multiple sclerosis patients modulate human T cells and exacerbate symptoms in mouse models. *Proc Natl Acad Sci U S A* 114**,** 10713-10718.

2. Jangi, S., Gandhi, R., Cox, L.M., Li, N., Von Glehn, F., Yan, R., Patel, B., Mazzola, M.A., Liu, S., Glanz, B.L., Cook, S., Tankou, S., Stuart, F., Melo, K., Nejad, P., Smith, K., Topçuolu, B.D., Holden, J., Kivisäkk, P., Chitnis, T., De Jager, P.L., Quintana, F.J., Gerber, G.K., Bry, L., and Weiner, H.L. (2016). Alterations of the human gut microbiome in multiple sclerosis. *Nature Communications* 7**,** 12015.

3. Berer, K., Gerdes, L.A., Cekanaviciute, E., Jia, X., Xiao, L., Xia, Z., Liu, C., Klotz, L., Stauffer, U., Baranzini, S.E., Kumpfel, T., Hohlfeld, R., Krishnamoorthy, G., and Wekerle, H. (2017). Gut microbiota from multiple sclerosis patients enables spontaneous autoimmune encephalomyelitis in mice. *Proc Natl Acad Sci U S A* 114**,** 10719-10724.

4. Tremlett, H., Fadrosh, D.W., Faruqi, A.A., Zhu, F., Hart, J., Roalstad, S., Graves, J., Lynch, S., Waubant, E., and Centers, T.U.N.O.P.M. (2016b). Gut microbiota in early pediatric multiple sclerosis: a case−control study. *European Journal of Neurology* 23**,** 1308-1321.

5. Miyake, S., Kim, S., Suda, W., Oshima, K., Nakamura, M., Matsuoka, T., Chihara, N., Tomita, A., Sato, W., Kim, S.W., Morita, H., Hattori, M., and Yamamura, T. (2015). Dysbiosis in the Gut Microbiota of Patients with Multiple Sclerosis, with a Striking Depletion of Species Belonging to Clostridia XIVa and IV Clusters. *PLoS One* 10**,** e0137429.

6. Wagley, S., Bokori-Brown, M., Morcrette, H., Malaspina, A., D'arcy, C., Gnanapavan, S., Lewis, N., Popoff, M.R., Raciborska, D., Nicholas, R., Turner, B., and Titball, R.W. (2019). Evidence of Clostridium perfringens epsilon toxin associated with multiple sclerosis. *Mult Scler* 25**,** 653-660.

7. Tremlett, H., Fadrosh, D.W., Faruqi, A.A., Hart, J., Roalstad, S., Graves, J., Lynch, S., and Waubant, E. (2016a). Gut microbiota composition and relapse risk in pediatric MS: A pilot study. *J Neurol Sci* 363**,** 153-157..

**Supplementary Table 5. Effects of treatment status in the abundances of the clades and genes with MS case-control discrepancies.**

| **Clade** | **Fold-change*^1^** | ***P*-value*^2^** |
| --- | --- | --- |
| *Erysipelatoclostridium sp.* | 0.81 | 0.76 |
| *Gemella morbillorum* | 0.50 | 0.59 |
| *Granulicatella* | 1.12 | 0.94 |
| *Granulicatella adiacens* | 1.12 | 0.82 |
| *Gabonia* | 1.93 | 0.47 |
| *Gabonia massiliensis* | 1.93 | 0.47 |
| *Sutterella sp.* | 121 | 0.26 |
| *Carnobacteriaceae* | 1.13 | 0.70 |
| **Gene** |  |  |
| Clo1100_2356 | 2.37 | 0.24 |
| Mahau_1952 | 1.67 | 0.74 |
| *^1^ Under treatment / Non treatment  *^2^ Wilcoxon rank sum test | | |

**URLs**

The URLs for data presented herein are as follows:

Trimmomatic, http://www.usadellab.org/cms/?page=trimmomatic

bowtie2, http://bowtie-bio.sourceforge.net/bowtie2/index.shtml

BMTagger, ftp://ftp.ncbi.nlm.nih.gov/pub/agarwala/bmtagger/

PRINSEQ, http://prinseq.sourceforge.net/

MEGAHIT, https://github.com/voutcn/megahit

MetaGeneMark, http://exon.gatech.edu/meta_gmhmmp.cgi

CD-HIT, http://weizhongli-lab.org/cd-hit/

KEGG genes, https://www.kegg.jp/kegg/genes.html

MGENES, ftp://ftp.genome.jp/pub/db/mgenes

DIAMOND, https://ab.inf.uni-tuebingen.de/software/diamond/

R car package, https://cran.r-project.org/web/packages/car/index.html

R glm2 package, https://cran.r-project.org/web/packages/glm2/index.html

R clusterProfiler package, http://bioconductor.org/packages/release/bioc/html/clusterProfiler.html

KEGG pathway, http://www.genome.jp/kegg/pathway.html

Pascal, https://www2.unil.ch/cbg/index.php?title=Pascal

R Hmisc package, https://cran.r-project.org/web/packages/Hmisc/index.html

R vegan package, https://cran.r-project.org/web/packages/vegan/index.html
